# Effect of Surface Texture on the Structural Adhesive Joining Properties of Aluminum 7075 and TEPEX^®^

**DOI:** 10.3390/ma15030887

**Published:** 2022-01-24

**Authors:** Alejandro Pereira, María Fenollera, Teresa Prado, Michal Wieczorowski

**Affiliations:** 1 Manufacturing Engineering Group (GEF) EEI Campus Lagoas, University of Vigo, 36310 Vigo, Spain; mfenollera@uvigo.es (M.F.); tprado@uvigo.es (T.P.); 2 Division of Metrology and Measurement Systems, Faculty of Mechanical Engineering, Institute of Mechanical Technology, Poznan University of Technology, Piotrowo Street 3, 60-965 Poznan, Poland; michal.wieczorowski@put.poznan.pl

**Keywords:** robotic polishing, surface metrology, aluminum 7075, TEPEX, structural adhesive

## Abstract

In the process of continuous improvement of manufacturing processes, this study was developed within the framework of the Ecovoss project, based on the inclusion of lightweight and new materials parts in the automotive sector. The objective was based on the replacement of aluminum welding operations with the option of adhesive operations with other types of materials such as polyamides or, in this case, a TEPEX^®^ composite material (Dynalite 202-c200/50% TYP 13). The aim of this work is to test the best texturing of substrate made in 7075 aluminum specimens manufactured by robotic polishing with an ABB 6640 robot. Another substrate is TEPEX composite. A structural adhesive film AF-163-2 from the 3M company (St Paul, MN, USA) is used, which must be applied according to the manufacturing procedure. The tests carried out are based on the topographic measurement of the surfaces to be joined with an Alicona focus variation microscope, and the uniaxial shear tests of adhesive samples have been analyzed. The texture of the surface failure has been analyzed, and the results confirm a significant correlation between the texture parameters of initial surfaces and maximum shear stress. The expected results should provide a better understanding of the surfaces aimed to optimize the adhesion of the studied materials.

## 1. Introduction

One of the basic objectives in aeronautics and in the automotive sector is to try to lower the overall weight in order to reduce CO_2_ emissions. Adhesive joining can be a very effective method to replace or complement traditional assembly and joining methods according to the specific need of the application. Advantages of adhesive bonding are overall structural lightening and reduced stress concentrations [1]. One of the disadvantages at an industrial level, when changing traditional assembly methods by adhesive, is based on the lack of knowledge regarding the behavior of adhesive joints over time. Therefore, studies are needed on the mechanical behavior of assemblies under extreme conditions, the effects of fatigue loads and the effects of the environmental conditions on the nominal static mechanical properties of the assembly. Uehara and Sakurai studied the influence of surface roughness on the joint strength of adhesion. They concluded that, for specific materials, there is an optimal surface roughness in the tensile strength of adhesion [2]. Adhesive joining is a preferred assembly technique for the fabrication of fiber reinforced composite metal hybrids. Khan et al. analyzed the behavior of the 7xxx series extruded aluminum alloy surface, treated with different conventional surface treatment methods and joined with fiberglass reinforced polymer (FRP). They observed that the surface treatment improves the adhesion between the aluminum and the epoxy compound [3]. Seo et al. evaluated the effect of surface treatments on the adhesive strength of polycarbonate aluminum joints. Adhesive strength shows a linear relationship with surface roughness and loading speed [4].

The influence of surface treatment techniques and conditions on single lap joint strength and interfacial properties has been investigated by Ozdemir et al. [5]. They implemented a numerical approach, which is a cohesive zone model, using ABAQUS™ in order to calculate a correlation between maximum interface traction and surface processing parameters, such as surface roughness and work of adhesion. Valdés et al. studied the multiobjective optimization on adhesive bonding of aluminum-carbon fiber laminate, from the multi-objective mathematical model point of view. They take into account the kind of adhesive, the overlapping of surface and surface finish (between acetone cleaning and plasma treatment) [6]. Trzepiecinski et al. studied the strength analysis of two types of fiber-metal laminates (FMLs), with a different way of preparing the adhesive coupling between 2024-T3 aluminum alloy sheets and a polymer/fiber layer. In this work, the 3M structural adhesive film AF 163-2 K is one of the used as an intermediate layer between prepreg and adherents. A significant increase in the FML peel strength was achieved with respect to the second FML variant, where coupling between adherents was produced using epoxy resin [7]. Ghumatkar et al. tested single strap joints with different adherents (mild steel and aluminum) bonded with an epoxy resin, and they found that an optimum surface roughness exists for a maximum bonding strength, and the roughness range depends on the adherent material [8].

Topographic analysis of surfaces is used in many different areas of human life and activity, among which conventional and unconventional machining processes [9], tribology [10], wear [11] and friction [12] are the most frequently mentioned. There are many different techniques for digitizing surface in micro scale [13], with different advanced options of extracting interesting features [14,15]. Surface topography influences on adhesion and related processes, including energy phenomena [16]. To make the bond between two surfaces stronger, they are often specially prepared in terms of the nature and size of the asperities. Sancaktar and Gomatam presented a study of the effect of surface roughness on adhesion for cold-rolled and hot-rolled steel surfaces followed by sandblasting and chemical etching [17]; Mizes presented the effect of surface roughness on fine particle adhesion [18], and Tayebi and Polycarpou presented the effect on adhesion and contact in microelectromechanical systems (MEMS) [19]. They all tried to find a relation between different features and strength. It has been shown that surfaces with high mean roughness height, excess and positive skewness in these applications are less susceptible to damage in the contact and near-contact zones. Méndez-Vilas et al. analyzed the effect of surface topography of latex and glass, on adhesion forces [20], while Prolongo et al. studied the effect of surface roughness of aluminum components bonded with epoxy adhesive on bonding strength [21]. The literature also provides examples of studies of the effect of wood surface roughness on soybean seed adhesion [22] and the effect of surface topography on the adhesion of human bone cells to inserted artificial parts [23]. Based on this, the research on adhesion of aluminum and TEPEX^®^ depending on surface topography was undertaken. The objective of the present research is to evaluate the influence of the topography by robotic polishing of aluminum 7075 in order to join with reinforced polymer TEPEX^®^ by means of 3M—AF163-2 structural adhesive.

## 2. Methodology

This research is a part of a project whose main objective is to replace an aluminum structural part with a composite one and consequently replace the welding process with the adhesive bonding, in order to improve process times and lighten the weight. As it was mentioned, this research consists in evaluating the strength of adhesion of two materials, by joining with an epoxy compound. 

**Figure 1:**
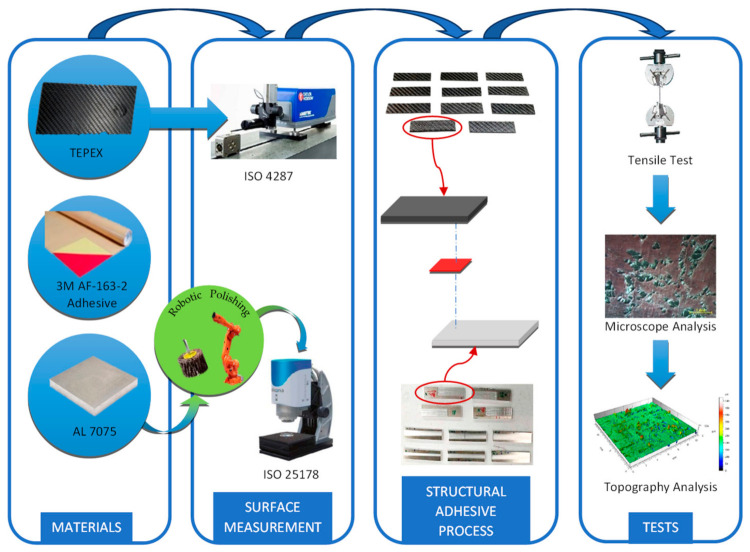
Methodology of experimental work.

As a first step in the methodology, the materials were selected. Aluminum 7075 was chosen as the base metal because there is extensive experience as a structural material in the aeronautical sector. In order to lighten the structures and increase energy efficiency, a TEPEX^®^ base was selected due to its higher tensile strength than aluminum. These materials have been selected due to are relatively easily accessible and generally used in industry. To subsequently be able to join the metal base with the composite, a structural adhesive film was used. Once the materials were selected, the surfaces were treated to measure their topography before adhesion between both bases. After the adhesion process, several tests were performed to analyze the bonding behavior. This methodology is shown in Figure 1. 

### 2.1. Materials

The materials that have been used to study the effect of the texture surface in the process of adhesive are:

- Aluminum 7075 T6. The characteristics of the 4 mm thick sheet are shown in Table 1.

TEPEX^®^ Dynalite 202 (laminar composite material of polyamide and 50% carbon fiber): Composite manufactured by “LANXESS Deutschland GmbH (Leverkusen, Germany)”, as shown in Table 2. Bond Laminates TEPEX^®^ Dynalite 202-C200/50 vol% (50 vol% carbon fiber in nylon 66) TYP 13.

- Structural Adhesive 3M AF-163-2 (Table 3): The structural adhesive film material is a thermosetting modified epoxy called AF163-2-K.06wt (carrying a PA66 polyamide mesh), from the 3M company. Some of its advantages are its high adhesion strength from -55 °C to 121 °C, high breaking strain and short curing time (60 min) at 120 °C. It should be kept at freezing temperature.

### 2.2. Processes

The TEPEX^®^ material has not been treated (surface was left unchanged), and the shape of the specimens was prepared simply for subsequent joining. However, the surface topography of this material was measured prior to sample preparation. The surface treatment of one of the materials, particularly the aluminum 7075, was made by polishing. To perform the surface treatment, a robotic polishing process was applied, consisting of the mechanical polishing of 4 mm 7075 aluminum sheet metal. An ABB (Zurich, CH) 6640 robot with a Peroni spindle with a rotation speed capacity of up to 60,000 rpm on a specially prepared stand was used to carry out the polishing operations. It is equipped with an ABB rotary table and a HSK-50 hydraulic tool clamping system [25]. The justification for having carried out the polishing treatment on robot has been the versatility, ease of adjustment and speed of the process, also taking into account the industrial applicability.

Polishing experiments of 7075 aluminum were performed with two types of tools in order to obtain a wide range of textures:

- Verox A280, dimensions Ø 40 mm × 20 mm(wide), 6 mm (support diameter) with a maximum rotational speed of 15,000 rpm and abrasiveness grade 280 (T1).
- Flap wheels, dimensions Ø 20 mm × 15 mm(wide), 6 mm (support diameter), with a maximum speed of 38,000 rev/min and a degree of abrasiveness of 60 (T2).

To plan the design of experiments (DOE) of polishing process, different fixed and variable conditions have been chosen [26]. As fixed process conditions, a constant feed rate of 1800 mm/min and a fixed tilt angle are assumed for each tool. As variable process conditions, the cutting speed (rpm) and the depth of cut were assumed. Two repetitions were carried out for each experiment. The recommendations given by the tool manufacturers regarding the maximum speeds were included. They were 15,000 rpm for the 40 mm diameter tool and 38,000 rpm for the 20 mm diameter tool [27]. Table 4 shows the designed DOE.

**Figure 2:**
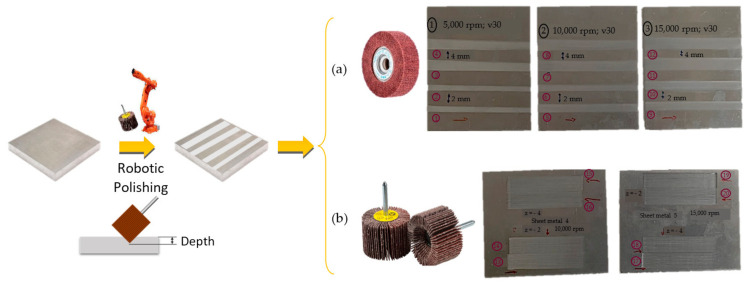
(**a**) 12 Samples polished with tool T1 Ø 40 mm, (**b**) 8 samples polished with tool T2 Ø 20 mm.

The different samples obtained are shown in the Figure 2.

**Figure 3:**
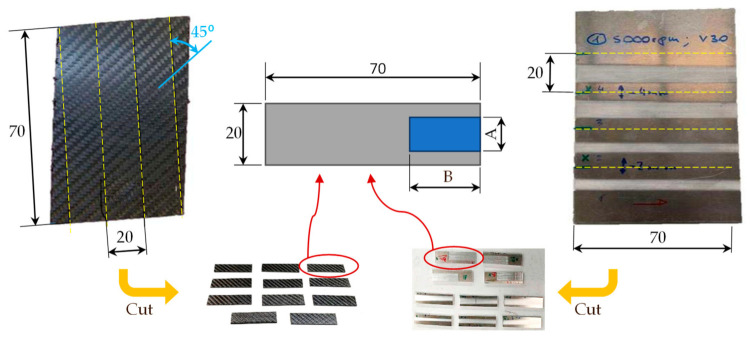
Marked of sheets of TEPEX and polished aluminum and final dimensions of samples for adhesive process.

The sheet metal and composite TEPEX^®^ were cut according to the dimensions of 70 mm × 20 mm, recommended by technical service of 3M, to make the tensile tests as it can be shown in Figure 3. 

To prepare the specimens, the available TEPEX^®^ sheets have been previously marked, taking into account 45° in the arrangement of the carbon fiber mesh. The aluminum sheets were also marked and cut with a band saw, taking into account the polished areas, as it can be seen in the previous figure. The main difficulty in cutting TEPEX^®^ composite is the positioning and clamping of the pieces, as well as the vibrations of the saw that could cause the fracture of the sheet due to the small thickness of the sample and the hardness of the material. The number of selected specimens of polished aluminum samples for the bonding process was reduced from initial 20, taking into account the dimensions specified for the adhesive film according to the minimal surface 11 mm × 23 mm (Table 5), recommended by 3M manufacturer. The samples 1, 6, 7, 9, 10, 11, and 17, have less than 11 mm of polished zone width and were therefore rejected, because in these samples the adhesive film (23 mm × 11 mm) would contact surface not polished.

**Figure 4:**
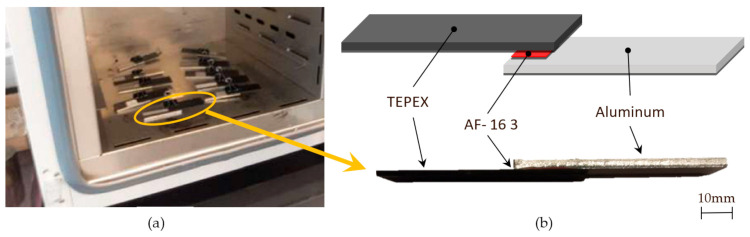
(**a**) Curing time in oven. (**b**) Sample prepared for tensile test.

The adhesive joining process consists of the following steps:

- Cleaning of surfaces to be bonded is done with isopropanol.
- Cutting the surface film 3M—AF-163-2 at rectangles of 23 mm × 11 mm.
- Separation of protective layers and manual bonding of surfaces with a pressure of 0.2 kg/cm^2^, made in manual press.
- Introduction into oven chamber with heating ramp at 120 °C, as it can be shown in Figure 4.
- Curing time: 60 min.
- Cooling time at room temperature 20 °C: 45 min.

### 2.3. Measurements

**Figure 5:**
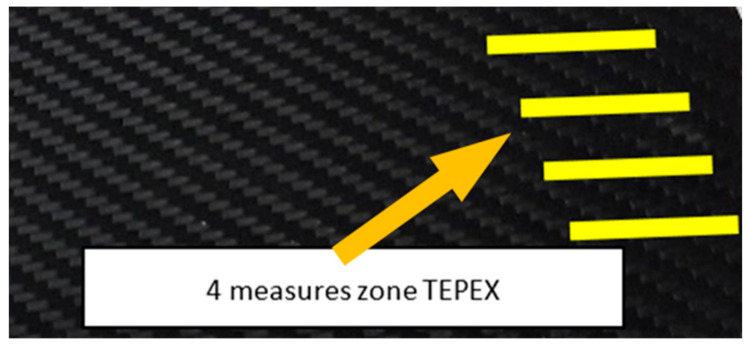
Roughness measurement of TEPEX^®^ surface.

**Figure 6:**
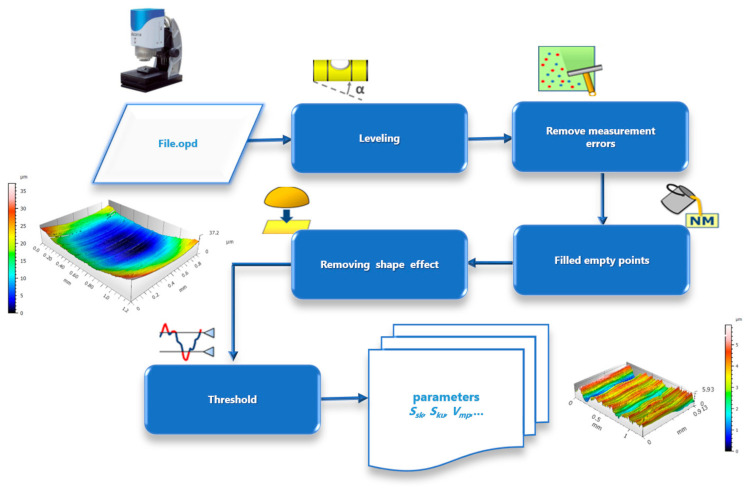
Methodology for data analysis of surface topography.

Different measurements were carried out depending on the surfaces, the TEPEX and the polishing tests:

- The TEPEX^®^ roughness, before cutting the specimens, has also been measured in four different areas (Figure 5) with a Taylor Hobson (Leicester, GB) profilometer, according to ISO-4287 [28]. The same direction and face have always been chosen to measure the profile of surface, and the cut-off equal to 0.8 mm has been selected. A measure length equal to 4 mm and a Gaussian filter have been used. The parameters used to define the roughness of the TEPEX^®^ material are shown in the Table 6. These measurements were obtained by the profile method, in order to know the initial parameters and their variability on the surface.
- The surface topography of the polished areas was measured using an Infinite Focus Alicona (Graz, Austria) focus variation microscope and further performed in order to obtain the surface parameters according to ISO 25178-2 [29]. The profiles obtained were then processed with MountainsMap surface data analysis software from Digital Surf (Besançon, France) [30]. Before obtaining the surface parameters, the data files obtained from the Alicona microscope were processed with this software to normalize surfaces and remove artifacts prior to analysis (leveling, removal of anomalous scan lines and shape effects or thresholding to remove spikes) such as shown in Figure 6. Measurements have been made on a surface over the polished area of 2 mm × 2 mm. The Gaussian standard filter was applied with a cut-off of 0.8 mm. The surface parameters shown in Table 7 have been chosen [31].

**Figure 7:**
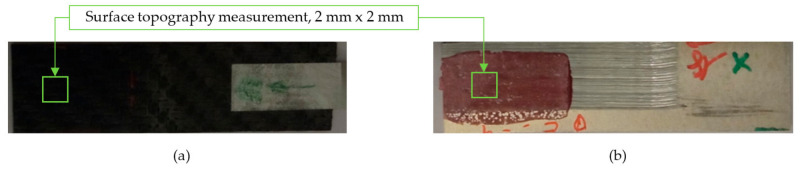
3D surface topography measurements of sample 18. (**a**) TEPEX side. (**b**) Aluminum side.

After the adhesive process, the shear tests were performed on a 30 kN Instron machine. After shearing the specimens, the following measurements were made:

- Visual surface analysis of specimens on a Nikon (Brighton, Michigan) SMZ800 optical microscope.
- Surface topography of adhesive fracture, on the two parts, with the same parameters selected in the polishing samples measurements (Figure 7).

## 3. Results and Discussion

### 3.1. Surface of TEPEX Substrate

The roughness measurements according to ISO 4287 of the TEPEX^®^ substrate were carried out with a Taylor Hobson profilometer. TEPEX measurements are simpler (2D) just to find whether the nature is random. 2D measurements were applied as the surface is not treated, and 3D topography inspection was performed after the tensile test. The obtained results for the parameters indicated in Table 6 are shown in Table 8.

The cleaning process is the unique treatment on the surface of the composite material. It can be seen that there are no significant differences in the selected areas which confirms a uniformity of the TEPEX^®^ substrate as shown in the mean value and deviation, for instance obtained for *Ra* parameter. There are significant deviations in the *Rt* parameter. This shows the presence of single high peaks and deep valleys that have a random character and do not influence the mean roughness of the surface. Moreover, skewness values (*Rsk*) demonstrate that in some cases peaks are dominant (e.g., TEPEX^®^ 1), while for others, the valleys play a more significant role (TEPEX^®^ 4). For kurtosis values (*Rku*), random character of surfaces is visible, particularly for TEPEX^®^ 3 and TEPEX^®^ 4 surfaces, with some deviations in the case of the two remaining ones. Nevertheless, average TEPEX^®^ with *Rsk* of 0.18 and *Rku* of 3.66 shows a quite stochastic character. 

### 3.2. Analysis of the Topography of Polishing Aluminum

The topography results of the measurements performed on the 7075 aluminum substrate are shown in Table 9. As expected, there are quantitative differences in the selection of the two polishing tools that provide considerable differences in the results of the topographic parameters. 

**Figure 8:**
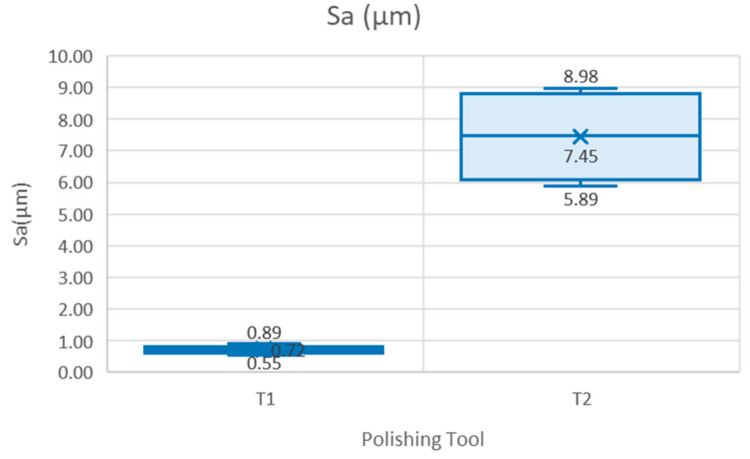
Parameter Sa versus polishing tool.

Figure 8 shows higher variability in the *Sa* results obtained with Tool 2, with Sa values between 5.9 µm and 11.4 µm and a mean of 7.45 µm. The range of Sa in the specimens machined with Tool 1 varies from 0.6 µm to 1.1 µm, with mean value of 0.7 µm. 

**Figure 9:**
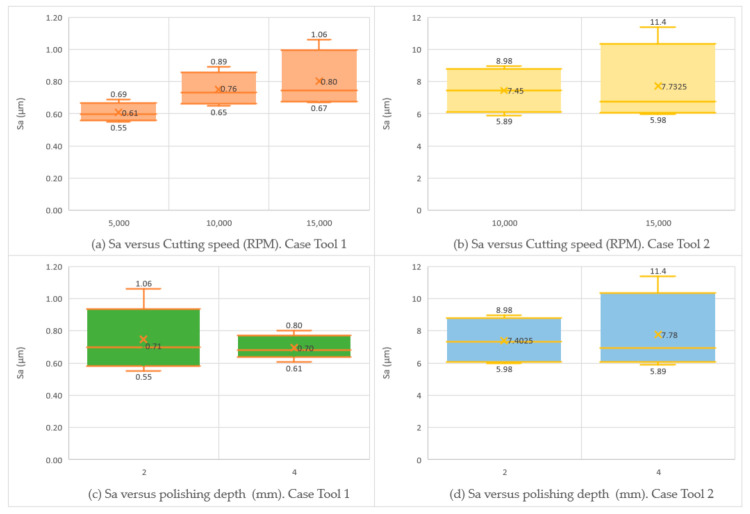
Value of Sa parameter versus polishing conditions (speed and depth) for Tool 1 (**a**,**c**) and for Tool 2 (**b**,**d**).

With regard to the surface polishing process conditions, no significant and clear correlations were observed between the selected variable parameters (cutting speed, measured in revolutions per minute of the spindle and polishing depth, measured in millimeters), and the measured surface parameters, particularly *Sa*. The data have been separated in relation to the tools. In the specific case of Tool 1 (Figure 9a), a slight increase in the parameter *Sa*, directly proportional to the rotational speed, can be observed. There is also an increase in the values as the rotation speed increases. In the case of Tool 2 (Figure 9b) an increase in the dispersion of the values as the rotational speed of the robot spindle increases can be noticed. The mean values of roughness are similar; *Sa* is equal to 7.5 µm for a spindle speed of 10,000 rpm and a mean of *Sa* = 7.7 µm in the case of spindle speed of 15,000 rpm. With regard to the depth of polishing (az parameter), the roughness values have also been analyzed for each tool. It can be seen in Figure 9c that in the case of *Sa* average values they remain the same at 0.7 µm, but a greater dispersion can be observed in the case of az = 2 mm. For Tool 2, Figure 9d, a slight increase in the *Sa* parameter, with respect to the depth was detected: *Sa*= 7.3 µm with az = 2 mm and *Sa* = 7.7 µm with az = 4 mm, with a higher dispersion in the case of az = 4 mm. It can be concluded that no significant correlations are found with respect to the cutting depth. Single peaks and valleys appear on the surfaces that can be seen from differences in *Sz* and *Sxp* values. These values, due to removing of aluminum particles, are dominant in relation to peaks. This can be observed from negative values of skewness (*Ssk*) for 19 out of 20 regions (the only positive skewness has a very low value above zero, which is 0.0356). Kurtosis close to 3 in most cases shows the random nature of polished samples. A different behavior of processes for Tool 1 and 2 can also be observed for *Sdq* and *Sdr* parameters. Slopes for Tool 2 are much steeper, which also affects in much larger developed interfacial area ratio.

### 3.3. Analysis of the Tensile Test

After measuring the topography of substrate and adhesive process, the tensile test was performed, and the results are shown in Table 10. The test was performed to see how strong the strength of the joint is and whether adhesion takes place on the surface. It should be noted that a total of 13 tests were performed for the AF-163-2 adhesive surface of 11 mm × 23 mm. It is also worth pointing out that the difficult fixing in the TEPEX^®^ substrate area, led to the inadequate positioning of some test specimens. 

There is a clamping problem corresponding to the TEPEX^®^ surface that requires manual tightening of the clamps, which leads to a torsional movement that should be avoided in the placement, due to the possible torsional breakage of join samples. This problem occurred in specimens 3 and 4, corresponding to texturing with Tool 1, and in specimens 13 and 16, corresponding to polishing with Tool 2.

**Figure 10:**
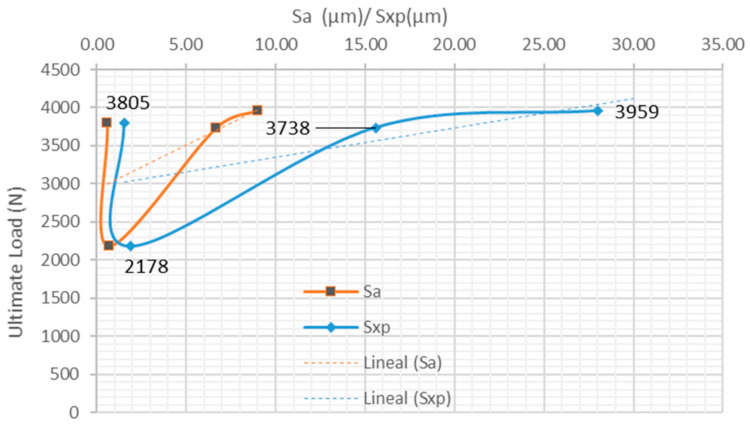
Behavior of ultimate load (N) versus *Sa/Sxp* (µm) of samples with clamping problem.

Analyzing the results with clamping problems, it is also observed that the higher the *Sa* parameter of polishing substrate, the higher the breaking load results are, except in the first sample 3 with lower *Sa*, as it can be shown in Figure 10. This trend can be seen in other parameters such as *Sz*, *Sv*, *Sp*, *Sq* and *Sxp*. The same result cannot be found for asymmetry and kurtosis parameters, since these parameters are not under a linear correlation. 

**Figure 11:**
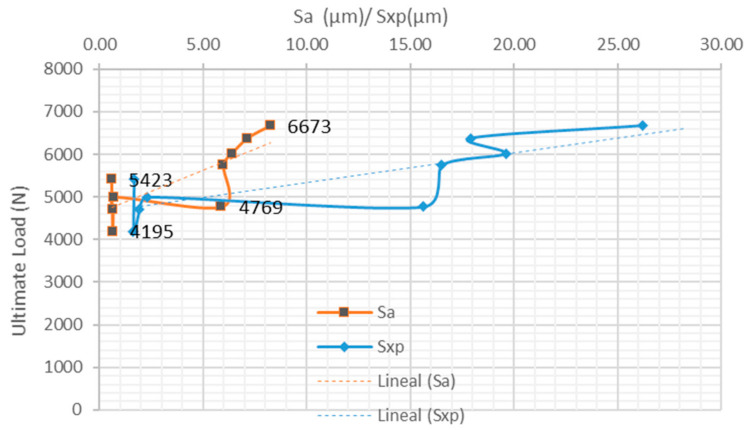
Behavior of ultimate load (N) versus *Sa/Sxp* (µm) of samples without clamping problem.

In the case of clamping problems, the highest proportion of adhesive rupture occurs in the bonding area of the TEPEX^®^ surface. It is observed that the maximum shear load reaches 3.959 N, which means that on the tested adhesive surface, the shear strength was 15.65 MPa. With regard to the specimens that have been correctly tested in tension, with clamping OK, the results obtained seem to follow the same trend. Figure 11 shows a trend where shear strength increases with increasing surface roughness. This is consistent with the works of Ghumatkar et al. [8] and Kalina et al. [32]. Obviously, the maximum shear load reached was higher than in the first case, with bad clamping. 

**Figure 12:**
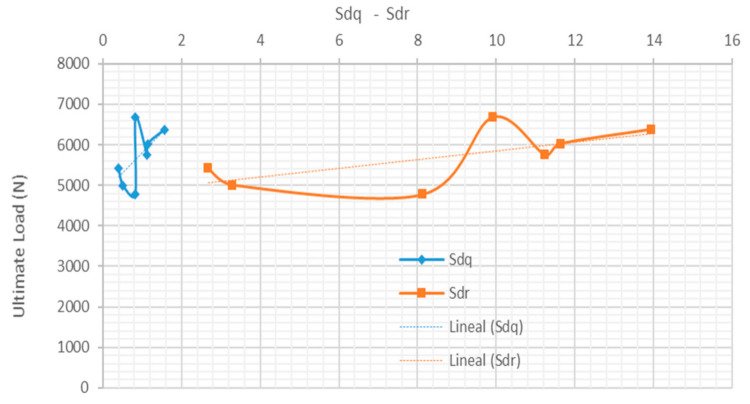
Behavior of ultimate load (N) versus *Sdq* and *Sdr* parameters.

Figure 12 represents the ultimate load versus the parameters *Sdq* and *Sdr*. This in turn corresponds to the research of Zielecki et al. [33]. The image shows that the increase of these surface parameters values is related to increase of the shear strength.

### 3.4. Examination of Fracture

**Figure 13:**
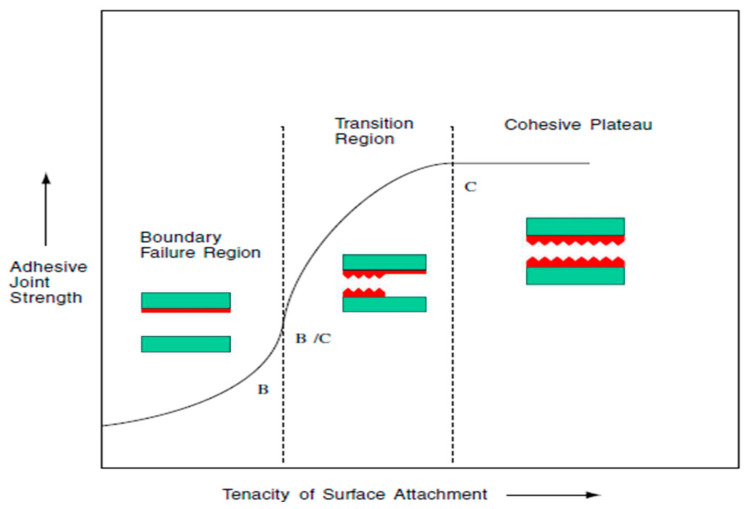
Schematic diagram of states in adhesion failures, B boundary failure, C cohesive failure and B/C mixed failure.

According to the theory of adhesives, there are four kinds of failure: boundary, substrate, cohesive and mixed. Three of them are shown in the Figure 13. In the case of the boundary failure region, the strength of interfacial contact is much less than the cohesive strength of the adhesive component. In the transition zone, the strength of the adhesive joint is sensitive to other parameters such as morphology of the surface, type of material, surface tension, the degree of interfacial surface attachment and environmental conditions. In the zone C, at a critical degree of surface fixation, the adhesive joint will rupture, showing a total cohesive fracture.

Table 11 shows the images of fractured surface in the samples 2 and 18, obtained with a Nikon SMZ 800 optical microscope. The sample 2 corresponds to an ultimate load equal to 5423 N, and the sample 18 corresponds to an ultimate load of 6379 N. In the TEPEX^®^ composite substrate, an area of remaining adhesive equal to 0.40 mm^2^ was measured. In the equivalent area measured on specimen 18, a larger adhesive area of 1.24 mm^2^ was observed. 

Analyzing the area on the aluminum substrate, it can be seen that the adhesive region appears in most cases. The measurement made in the adhesive-free region in sample 2 resulted in a surface area of 0.68 mm^2^. In the case of sample 18, the area of boundary failure is equal to 3.83 mm^2^. It can be concluded that a mixed failure occurred in these tested specimens, meaning a cohesive and boundary failure. It would be interesting to study in future works the proportion of boundary/cohesive failure and improve the surface attachment with the TEPEX^®^ substrate, in order to obtain the zone where the boundary failure would be lower. The results are in agreement with the two failure modes A and B studied by Kumar et al. [34]. They tested under uniaxial tensile loading adhesively bonded scarf joints, comprising unidirectional carbon fiber-reinforced epoxy adherents and AF-163-2 film adhesive with 0.15 mm thickness. Their results revealed that failure occurred into two modes, namely, fiber fracture and pull-out (Mode A) and cohesive shear failure of the adhesive film (Mode B).

The 3D topographic images of the same specimens analyzed above, sample 2 and sample 18, are shown in Table 12. Firstly, the difference between the polishing of the aluminum substrate of sample 2 (*Sa* = 0.59) and sample 18 with high anisotropy and directionality (*Sa* = 7.15) can be clearly seen.

The AF163 adhesive mostly adheres to the aluminum, and on the observed specimens, the adhesive layer is thicker in specimen 18, which may be due to a greater ultimate load. Traces of adhesive can be seen on both substrates, but more can be detected on the aluminum than on the TEPEX^®^ composite material. This determines that there is a mixed fracture. Adhesive fractures can also be seen mainly in the area of the TEPEX^®^ substrate, which corresponds to the topography prior to bonding. In the region of the aluminum substrate, a much smaller area of boundary fracture is observed, which would imply a lower proportion of adhesive breakage. The parameters obtained in the failure zone for the samples 2 and 18 are shown in Table 13.

It can be noticed that there is a change of the average surface in the case of the aluminum substrate. The increase in the average roughness is probably due to the higher amount of adhesive in the aluminum area. The difference of *Sa* in the TEPEX^®^ substrate between fracture samples is smaller than in aluminum fracture because the roughness in the substrate was similar. However, there is a slight increase in *Sa* between TEPEX^®^ zone and fracture TEPEX^®^ zone, and this indicates that a mixed failure occurs, with a lower proportion of cohesive fracture. This is in agreement with the results of Kumar et al. [23].

## 4. Conclusions

In this research work, we have studied the relationship between the topography of a structural material bonded with another one in order to form a structural part of a vehicle. The selected materials are oriented to reduce the weight of the vehicle, taking into account that the project is part of a framework project to increase energy efficiency in the automotive sector. Aluminum 7075 and a composite material called TEPEX^®^ have been chosen for their high tensile stress values. Taking into account the poor weldability of aluminum 7075, the welding process was replaced with a structural adhesive process and a 3M film adhesive, AF163-2, was selected. A study of the robotic polishing process was also carried out in order to vary the topography of the material and apply the process at industrial level. In this robotic polishing of aluminum 7075, different process conditions have been experimented, such as tools, cutting speeds (rpm), feed rates and polishing depth, in order to obtain a variability of the topography of the surface obtained. The study of the process conditions in relation to the topography obtained in aluminum 7075 was an initial objective. The main objective was to study, from the point of view of surface metrology, the effect of the topography of aluminum 7075 on the maximum shear stress in the bonded assembly, formed by TEPEX^®^ and aluminum 7075.

To summarize the results analyzed, the following conclusions can be drawn:

- The cutting conditions and polishing feed rate do not significantly influence the results of the topography of the substrate aluminum 7075. A slight increase in *Sa* occurs with both tool T1 and tool T2 as cutting speed increases. There is no significant correlation between *Sa* and the increment of polishing depth.
- The variability of the topography of the aluminum substrate, taking the *Sa* parameter as a reference, is mainly determined by the type of tool used. The *Sa* range in the case of tool T1 is 0.4 µm and the *Sa* range in the case of tool T2 is 3.1 µm. The same behavior occurs with the *Sxp* parameter.
- A slight correlation between the *Sa* values of the aluminum substrate and the ultimate load can be observed. The correlation between *Sdq* and *Sdr* with the shear strength is higher than *Sa* and *Sxp*.
- The analysis of the surfaces tested at failure zone determines that there is adhesive material on both substrates, so that a mixed fracture is produced, although it should be noted that there are areas of adhesive fracture, especially on the TEPEX^®^ substrate. The lower is the adhesive fracture, the higher is the shear strength. The realization of new experiments consisting in modifying the texture of TEPEX^®^ in the same way as aluminum is a future line of action in order to increase the breaking strain of the bonded part.
- From the productivity point of view, it would be interesting to carry out the process at high speeds to reduce the processing time. Robotic polishing is a very fast and easy to apply operation, from the industrial point of view.

## Figures and Tables

**Table 1:** Properties of aluminum 7075 T6 (Data from [24]).

**Chemical Composition (wt %)**	Zn	Mg	Cu	Mn	Fe	Cr	Al
	5.24	2.23	1.41	0.09	0.21	0.24	Bal.
**Mechanical Properties**	Tensile Strength				Elongation		
	572 MPa				14%		

**Table 2:** TEPEX^®^ Dynalite 202 characteristics (Data Sheet Lanxess).

Fiber	Matrix	Mechanical Properties	
Carbon	PA66	Tensile Strength	Elongation
50%	50%	700 MPa	1.5%

**Table 3:** Characteristics 3M™ Scotch-Weld™Structural Adhesive Film AF 163-2 (Technical Data sheet 3M).

Adhesive Type	Cure Temperature	Cure Time	Ultimate Shear (23 °C)	Observations
Epoxy	120 °C	60 min	47.9 MPa	High fracture toughness and peel strength

**Table 4:** Design of experiments of polishing process.

Number Test	Tools	Feed (mm/min)	Cutting Speed (rpm)	Depth (mm)
12	T1 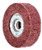	1800	5000	2 & 4
			10,000	
			15,000	
8	T2 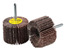	1800	10,000	2 & 4
			15,000	

**Table 5:** Samples selected with polishing zone A ≥ 11 mm and B ≥ 23 mm.

Polishing Sample A7075	A ≥ 11 mm 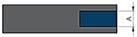	B ≥ 23 mm 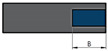
2	11	35.8
3	11	25.0
4	11	25.0
5	11	31.2
8	11	23.8
12	11	23.5
13	12	24.3
14	15	26.7
15	14	25.5
16	13	24.3
18	15	26.5
19	14	24.2
20	14	24.9

**Table 6:** Parameters profile TEPEX^®^ surface according to ISO 4287.

Parameter	Description	Expression	
*Ra* (µm)	Arithmetic mean profile height	$Ra=\frac{1}{L}\int_{i}^{x}\left|Z_{i}\right|\times dx$	(1)
*Rq* (µm)	Profile mean square height	$Rq=\sqrt{\frac{1}{L}\int_{i}^{x}Z^{2}\times dx}$	(2)
*Rt* (µm)	Maximum height over the measured profile	Rt=Rp+Rv	(3)
*Rsk*	Skewness	Rsk=1Rq31L∫ixZ3×dx	(4)
*Rku*	Kurtosis	Rku=1Rq41L∫ixZ4×dx	(5)

**Table 7:** 3D Surface topography parameters (ISO 25178-2).

	Description	Expression	
*Sa* (µm)	Arithmetic mean surface height	$S_{a}=\frac{1}{m\times n}\overset{m}{\underset{0}{\int }}\overset{n}{\underset{0}{\int }}\left|Z\left(x,y\right)\right|dxdy$	(6)
*Sq* (µm)	Mean square surface height	$S_{q}=\frac{1}{m\times n}\sqrt{\overset{m}{\underset{0}{\int }}\overset{n}{\underset{0}{\int }}Z^{2}\left(x,y\right)dxdy}$	(7)
*Ssk*	Surface asymmetry. Dimensionless	$S_{sk}=\frac{1}{m\times n\times S_{q}^{3}}\overset{m}{\underset{0}{\int }}\overset{n}{\underset{0}{\int }}Z{\left(x,y\right)}^{3}dxdy$	(8)
*Sku*	Surface kurtosis. Dimensionless	$S_{ku}=\frac{1}{m\times n\times S_{q}^{4}}\overset{m}{\underset{0}{\int }}\overset{n}{\underset{0}{\int }}Z{\left(x,y\right)}^{4}dxdy$	(9)
*Sxp*	The Maximum Peak Height, Sxp (p, q)	Measure of the difference in surface heights from the value of the area material ratio of “p” and the area material ratio of “q”. The default value for “p” is 97.5% and for “q” is 50%.	
*Sdq*	The surface gradient. RMS slope	$S_{dq}=\sqrt{\frac{1}{mxn}\overset{m}{\underset{0}{\int }}\overset{n}{\underset{0}{\int }}\left[{\left(\frac{\partial z\left(x,y\right)}{\partial x}\right)}^{2}+{\left(\frac{\partial z\left(x,y\right)}{\partial y}\right)}^{2}\right]dxdy}$	(10)
*Sdr*	The developed interfacial area ratio	$S_{dr}=\frac{1}{mxn}\left[\overset{m}{\underset{0}{\int }}\overset{n}{\underset{0}{\int }}\left(\sqrt{\left[1+{\left(\frac{\partial z\left(x,y\right)}{\partial x}\right)}^{2}+{\left(\frac{\partial z\left(x,y\right)}{\partial y}\right)}^{2}\right]-1}\right)dxdy\right]$	(11)

**Table 8:** Results of measurement roughness of TEPEX surface.

Results TEPEX	*Ra* (µm)	*Rt* (µm)	*Rq* (µm)	*Rsk*	*Rku*
Zone TEPEX^®^ 1	4.1	67.5	5.78	0.77	4.67
Zone TEPEX^®^ 2	3.55	32.23	4.68	0.33	4.26
Zone TEPEX^®^ 3	3.65	25.97	4.52	0.08	2.82
Zone TEPEX^®^ 4	3.74	25.8	4.48	−0.47	2.89
Average TEPEX^®^	3.76	37.88	4.87	0.18	3.66
Standard deviation(σ)	0.24	19.98	0.62	0.52	0.94

**Table 9:** Surface topography of polishing aluminum 7075 substrate.

Sample	Tool	*Sa* (μm)	*Sv* (μm)	*Sp* (μm)	*Sz* (μm)	*Sq* (μm)	*Ssk*	*Sku*	*Sxp* (μm)	*Sdq*	*Sdr* %
1	T1	0.551	3.25	3.17	6.43	0.629	−0.189	3.06	1.45	0.18	1.17
2	T1	0.59	3.61	4.01	7.61	0.747	−0.284	3.42	1.66	0.186	1.174
3	T1	0.606	3.99	5.11	9.1	0.755	−0.107	2.88	1.53	0.186	1.167
4	T1	0.688	3.96	4.7	8.66	0.864	−0.269	3.1	1.87	0.2375	1.602
5	T1	0.706	2.89	3.99	6.89	0.91	−0.651	3.58	2.3	0.2594	1.827
6	T1	0.891	5.24	4.34	9.58	1.13	−0.712	3.67	2.68	0.525	3.636
7	T1	0.759	4.18	3.98	8.16	0.95	−0.44	3.13	2.17	0.2068	1.379
8	T1	0.648	3.24	3.84	7.07	0.824	−0.043	3.16	1.65	0.389	2.67
9	T1	1.06	7.56	5.87	13.5	1.49	−0.808	5.58	4.29	0.2801	1.971
10	T1	0.687	4.09	2.83	6.92	0.888	−0.653	3.72	2.23	0.1776	1.133
11	T1	0.802	4.06	3.92	7.98	1	−0.071	2.93	2.03	0.22	1.576
12	T1	0.671	4.75	3.29	8.03	0.85	−0.447	3.39	1.93	0.5	3.304
13	T2	8.98	36.5	24.3	60.8	11	−0.686	2.85	28	0.685	7.66
14	T2	8.25	33	19.3	52.4	10.2	−0.825	3.11	26.2	0.816	8.129
15	T2	5.89	24	21.2	45.2	7.27	−0.329	2.67	15.6	0.8253	9.912
16	T2	6.68	26.1	20.1	46.2	8.03	−0.231	2.62	15.6	1.122	11.25
17	T2	11.4	66.5	47.3	114	14.7	−0.603	3.13	37.9	1.412	17.88
18	T2	7.15	27.1	23.9	50.9	9.09	0.0356	2.64	17.9	1.126	13
19	T2	6.4	39	17.7	56.7	8.27	−0.935	5.05	19.6	1.14	11.64
20	T2	5.98	25.4	20.6	46	7.62	−0.115	3.04	16.5	1.568	13.95

**Table 10:** Results of tensile test.

Sample	Tool	Comments	Ultimate Load (N)	Ultimate Shear (Mpa)
2	T1	ok	5423	21.43
3	T1	ko clamping	3805	15.04
4	T1	ko clamping	2178	8.61
5	T1	ok	5002	19.77
8	T1	ok	4195	16.58
12	T1	ok	4712	18.62
13	T2	ko clamping	3959	15.65
14	T2	ok	6673	26.38
15	T2	ok	4769	18.85
16	T2	ko clamping	3738	14.77
18	T2	ok	6379	25.21
19	T2	ok	6023	23.81
20	T2	ok	5744	22.70

**Table 11:** Failure modes of sample 2 and 18.

Sample		TEPEX^®^ 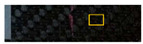	AA7075 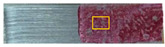
2	Before Failure	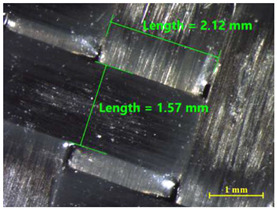	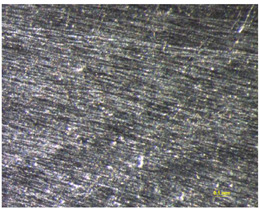
2	After Failure	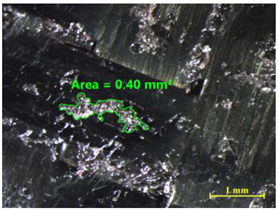	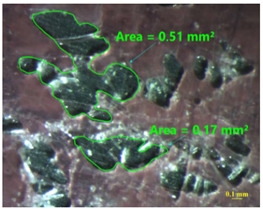
18	Before Failure	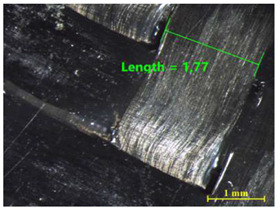	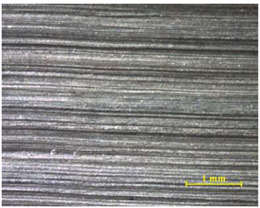
18	After Failure	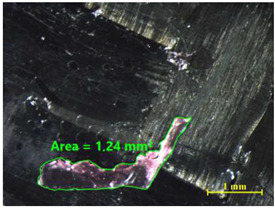	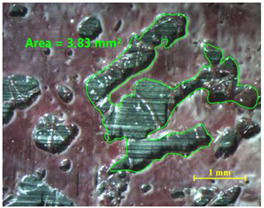

**Table 12:** 3D topography of samples 2 and 18.

Sample		TEPEX^®^ 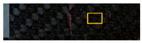	AA7075 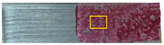
2	Before Failure	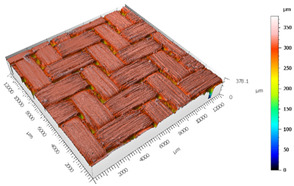	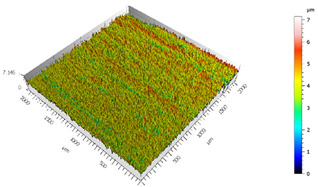
2	After Failure	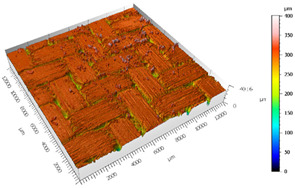	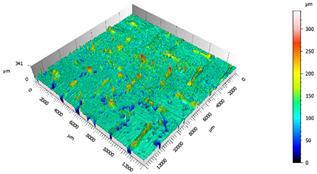
18	Before Failure	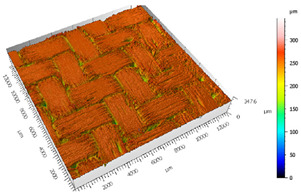	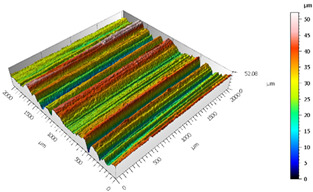
18	After Failure	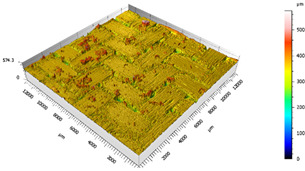	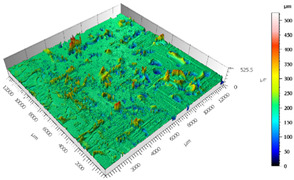

**Table 13:** Surface parameter in the samples 2 and 18 of fracture zone.

Zone	Sample 2		Sample 18			
	*Sa* (μm)	*Sdq*	*Sdr*	*Sa* (μm)	*Sdq*	*Sdr*
Aluminum	0.47	0.12	0.72%	6.81	0.32	4.63%
TEPEX (3D)	8.92	0.50	7.08%	9.18	0.54	7.4%
Fracture Aluminum	16.87	1.03	19.84%	28.49	1.464	29.1%
Fracture TEPEX(3D)	11.65	0.65	11.31%	11.27	0.81	12.57%

## Data Availability

Data are contained within the article.
